# Advanced maternal age pregnancy and its adverse obstetrical and perinatal outcomes in Ayder comprehensive specialized hospital, Northern Ethiopia, 2017: a comparative cross-sectional study

**DOI:** 10.1186/s12884-020-2740-6

**Published:** 2020-01-30

**Authors:** Mihret-ab Mehari, Hayat Maeruf, Carmen C. Robles, Solomon Woldemariam, Tesfay Adhena, Mussie Mulugeta, Abera Haftu, Hadgay Hagose, Henok Kumsa

**Affiliations:** 1 Mekelle University, College of Health Sciences, P.O. Box 1871, Mekelle, Ethiopia; 2 Dr. Tewelde Legesse Health Sciences College, P.O. Box 306, Mekelle, Ethiopia; 3 Axum University, Axum, Ethiopia; 4 Woldiya University, Woldiya, Ethiopia

**Keywords:** Advanced maternal age pregnancy, Adverse perinatal outcomes, Adverse obstetrical outcomes, Ayder comprehensive specialized hospital

## Abstract

**Background:**

Advanced maternal age generally denotes age after 35 years during the time of delivery. Despite the fact that being pregnant at any reproductive age is not risk-free, older gravidity usually culminates with adverse outcomes both to the mother and fetus or neonate. This study aimed to determine the association of adverse obstetrical and perinatal outcomes with advanced maternal age pregnancy. The study was conducted in Ayder comprehensive specialized hospital, north Ethiopia, from 2015 to 2017.

**Methods:**

chart review comparative cross-sectional study was employed. Data were retrieved from medical charts of 752 pregnant mothers (376 each for both the study;> 35-year-old and reference group;20-34 year old). Data was collected using a pretested and structured checklist using systematic sampling and data was entered & analyzed using SPSS version 20. Binary and multivariable logistic regression was run to determine the association of independent variables with dependent variables.

**Results:**

This study revealed that advanced maternal age pregnancy was significantly associated with pregnancy induced hypertension [AOR 4.15, 95% CI (2.272–7.575), *p* <  0.001], ante partum hemorrhage [AOR 2.54, 95% CI (1.32–4.91), *P* = 0.005] & cesarean delivery [AOR 2.722, 95% CI (1.777–4.170), *p* <  0.001]. Furthermore, advanced maternal age pregnancy was also increasingly associated with adverse perinatal outcomes like preterm delivery [AOR 3.622, 95% CI (1.469–8.930), *p* = 0.005], low birth weight **[**AOR 3.137, 95% CI (1.324–7.433), *p* = 0.009], perinatal death [AOR 2.54, 95% CI (1.141–5.635), *p* = 0.022] and low fifth minute APGAR score [AOR 7.507, 95% CI (3.134–17.98), p <  0.001]. Notwithstanding this, maternal age was not found to be associated with amniotic fluid disturbances, premature rupture of membranes and post-term pregnancy.

**Conclusions:**

Advanced maternal age is markedly linked with adverse obstetrical and perinatal outcomes. Therefore, it is better for health care providers to counsel couples, who seek to have a child in their later ages, about the risks of advanced maternal age pregnancy. In addition, health care workers need to emphasize on how to improve advanced age mothers’ health through the utilization of contraception to reduce pregnancy in this age group.

## Introduction

Advanced maternal age is usually defined as being 35 years or older which is believed to predispose mothers to enormous adverse outcomes during pregnancy [1]. A study done in 29 countries (Africa, Asia, Middle East, and Latin America) revealed that the magnitude of pregnant women with advanced maternal age was 12.3% [2]. A previous study conducted in the United States of America showed that the average age of women at first birth has consistently increased over the last four decades, with the birth rate for women aged 40–44 more than doubling from 1990 to 2012 [3]. A retrospective comparative study done in a South Africa tertiary hospital revealed that the prevalence of advanced maternal age was 17.5% [4].

Advanced maternal age is associated with various economic, social and health complications to the mother and to the fetus or neonate as well [2, 5]. Various studies conducted on the topic elucidated that the aftermath of advanced maternal age (AMA) is highly linked with the occurrence of pregnancy-induced hypertension, DM, maternal near-miss, increased cesarean delivery, malpresentation, and maternal death. Alongside with this, AMA also results in neonatal complications, such as Low Apgar score, NICU admission, preterm delivery, low birth weight, birth defects, chromosomal abnormalities and perinatal death [2, 4–7].

Fertility in women starts to decline in the early thirties and even decreases faster after mid and late thirties. Women with advanced age usually have a relatively lower tendency to achieve pregnancy within a short period. The probability of achieving pregnancy in a single menstrual cycle, fecundability, is decreased in these age groups [8, 9].

Globally, the academic community has extensively explored the association of AMA with adverse obstetrical and perinatal outcomes. However, few researches have been conducted to identify the association of AMA with these outcomes in the study area and in Ethiopia. Hence, there is a need to carry out research to determine the magnitude of obstetrical and perinatal outcomes associated with AMA. On the other hand, identifying the outcomes of AMA pregnancy will be useful in designing effective sensitization programs for couples and empowering them about informed choices for pregnancies during advanced maternal age. Furthermore, the result of this study can be utilized by concerned bodies to optimize natal care given for advanced aged mothers.

## Methods

A chart review retrospective comparative cross-sectional study was conducted from November 2017 to December 2017 to assess the association of advanced maternal age pregnancy with adverse obstetrical and perinatal outcomes in Ayder Comprehensive Specialized Hospital. The hospital is one of the biggest tertiary and teaching hospitals with a good reputation in the country. It is located in Mekelle city, 783 km north of Addis Ababa, the capital city of Ethiopia. The hospital has 500 beds and serves more than 17,000 patients every year. It is estimated that around 3000 women give birth annually in the hospital.

The sample size was calculated using Open Epi Data statistical software. A one to one ratio of study to reference group, 95% confidence level and power of 90% was assumed. Taking cesarean delivery rate among exposed which is 27 and 17% among non - exposed [10], it yielded a total sample size of 752 (376 charts of mothers whose age is > 35 years and 376 charts of mothers whose age is between 20 to 34 years). Charts of mothers who gave birth in Ayder Comprehensive Specialized Hospital from September 2015 to September 2017 which were selected using systematic sampling were reviewed in this study. Pregnant mothers whose age is ≥35 years were regarded as a study group and mothers whose age is between 20 and 34 years were taken as a reference group. Mothers with pre-existing medical disease, twin pregnancy, Rh negative mothers and incomplete records were excluded from the study.

Data were retrieved from the sampled mothers’ chart using a structured checklist which was developed after reviewing variables discussed in various literature to enable the researchers to collect data on socio-demographic status, obstetric history, mode of delivery, adverse obstetrical and perinatal outcomes.

Prior to the data collection, the checklist was reviewed by senior researchers for its validity. Four graduate midwives with previous experience in data collection were hired as data collectors and two MSc in Clinical Midwifery students were recruited as supervisors. Three days of training on data collection was provided. Ten percent of the collected data was checked by supervisors every day for its completeness and the principal investigator monitored the overall tasks. The checklist was pretested in 10% of the calculated sample size in the same hospital on charts of mothers who visited this hospital four years prior to the study period. After this pretest was undertaken, one outcome variable (NICU admission) and one explanatory variable (educational status) were omitted from the checklist.

The data were checked for completeness, then coded, entered, and analyzed using Statistical Package for Social Sciences (SPSS) version 20. Descriptive statistics were used to compute frequency, percentile, mean and median of different variables. A Binary regression model was employed to test the association between the dependent and independent variables. Dependent variables are adverse obstetrical outcomes such as PIH, APH, cesarean delivery, amniotic fluid disorders, PROM, GDM, and postpartum hemorrhage. Moreover, perinatal outcomes such as preterm delivery, low birth weight, low fifth minute Apgar score, post-term pregnancy, and perinatal death are dependent variables. Each adverse obstetrical outcome was assessed if it has made a significant association with maternal age, residence, gravidity, number of ANC visits, adverse obstetric history and malpresentation. On the other side, each perinatal outcome was tested to see its association with PIH, APH, amniotic fluid disorders & PROM, alongside with maternal age, residence, gravidity, number of ANC visits, bad obstetric history and malpresentation. All variables with *P*-value ≤0.25 were included in the multivariable analysis. The magnitude of the association was measured using odds ratio at 95% confidence interval and statistical significance was declared at *P*-value of ≤0.05.

## Results

### Socio-demographic and obstetric characteristics of study participants

A total of 752 charts of mothers were reviewed in this study. The median age of the study group (≥ 35 years old) was found to be 37 years with (IQR of 35–40 years) whereas the mean age of the reference group (20–34 years old) was 26.6 years with a standard deviation of 3.5 years. Almost one fifth (19.1%) of advanced maternal age mothers in this study had ANC follow up of less than four times. Nearly half of adult pregnant mothers (43.1%) were pregnant for the first time. The magnitude of malpresentation among advanced age mothers and adult mothers was 9.6 and 4.3%, respectively. Socio-demographic and obstetric characteristics of study participants are listed in Table 1.

**Table 1:** Distribution of obstetrics characteristics & residence of advanced and adult mothers, Ayder Comprehensive Specialized Hospital, Northern Ethiopia, 2017/18 (*n* = 752)

Obstetrics characteristics		Maternal age					
		20–34 years		35+ years		Total	
		*N*	%	*N*	%	*N*	%
Residence	Rural	105	27.9	154	41.0	259	34.4
	Urban	271	72.1	222	59.0	493	65.6
Adverse Obstetrics History	Yes	20	5.3	37	9.8	57	7.6
	No	356	94.7	339	90.2	695	92.4
Number of ANC Visits	less than four visits	46	12.2	72	19.1	118	15.7
	four and above visits	330	87.8	304	80.9	634	84.3
Malpresentation	No	360	95.7	340	90.4	700	93.1
	Yes	16	4.3	36	9.6	52	6.9
Gravidity	Primigravida	162	43.1	23	6.1	185	24.6
	Multigravida	202	53.7	205	54.5	407	54.1
	Grand multigravida	12	3.2	119	31.6	131	17.4
	Great grand multigravida	0	0.0	29	7.7	29	3.9

### Magnitude of adverse obstetrical outcomes

**Fig. 1:**
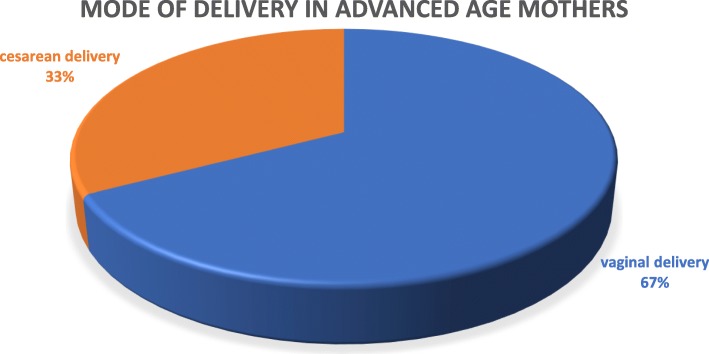
proportion of mode of delivery in advanced age mothers, Ayder Comprehensive Specialized Hospital, Northern Ethiopia, 2017/18 (*n* = 376)

**Fig. 2:**
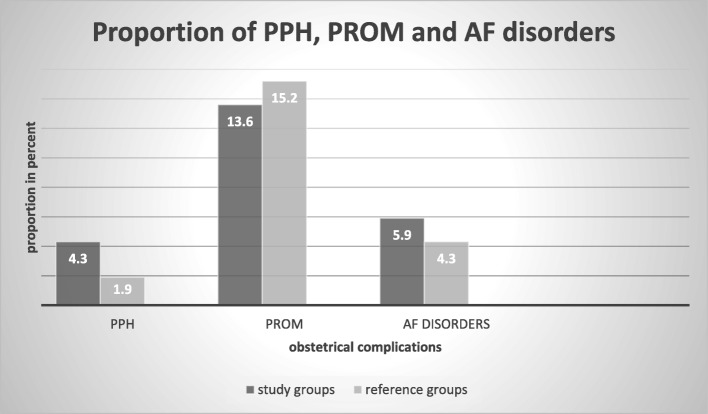
comparison of proportion of PPH, PROM and AF disorders between study and reference group, Ayder Comprehensive Specialized Hospital, Northern Ethiopia, 2017/18 (*n* = 752)

This study revealed that the magnitude of pregnancy-induced hypertension and antepartum hemorrhage was 87 (11.6%) and 76 (10.1%), respectively. There were only 3 (0.8%) adult pregnant mothers whose pregnancies were complicated by gestational DM which is almost invariable from their advanced age counterparts, 6(1.8%). Nearly one third (32.1%) of advanced age mothers underwent cesarean section which is higher than adult mothers, 19.4%. Overall about a quarter of mothers (26.1%) in this study gave birth via cesarean section (shown in Fig. 1). Figure 2 shows the proportion of PPH, PROM and amniotic fluid disorders among the study group and reference group. The magnitude of overall adverse obstetrical outcomes among study group and reference group is presented in Table 2.

**Table 2:** Magnitude of adverse obstetrical outcomes of advanced and adult mothers, Ayder Comprehensive Specialized Hospital, Northern Ethiopia, 2017/18 (*n* = 752)

Obstetrical Outcomes		Maternal age					
		20–34 years		35+ years		Total	
		*N*	%	*N*	%	*N*	%
Pregnancy Induced Hypertension	No	355	94.4	310	82.4	665	88.4
	Yes	21	5.6	66	17.6	87	11.6
Gestational Diabetes Mellitus	No	373	99.2	370	98.4	743	98.8
	Yes	3	0.8	6	1.6	9	1.2
Antepartum Hemorrhage	No	358	95.2	318	84.6	676	89.9
	Yes	18	4.8	58	15.4	76	10.1
Amniotic Fluid Disorders	No	360	95.7	354	94.1	714	94.9
	Yes	16	4.3	22	5.9	38	5.1
Premature Rupture of Membrane	No	319	84.8	325	86.4	644	85.6
	Yes	57	15.2	51	13.6	108	14.4
Mode of Delivery	Vaginal Delivery	303	80.6	253	67.3	556	73.9
	Cesarean Delivery	73	19.4	123	32.7	196	26.1
Postpartum Hemorrhage	No	369	98.1	360	95.7	729	96.9
	Yes	7	1.9	16	4.3	23	3.1

### Magnitude of adverse perinatal outcomes

**Fig. 3:**
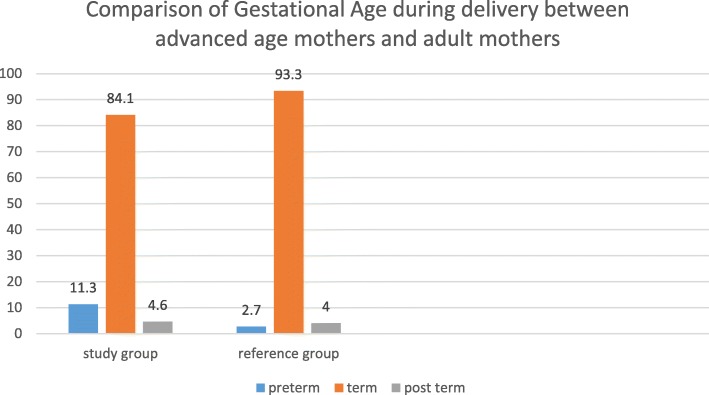
comparison of gestational age at the time of delivery between advanced age mothers and adult mothers, Ayder Comprehensive Specialized Hospital, Northern Ethiopia, 2017/18 (*n* = 752)

There were 50 perinatal deaths documented in this study nine (18%) of which were intrauterine fetal death, four (8%) were early neonatal death and the remaining three-fourths of perinatal deaths occurred during the intrapartum period (stillbirth). The mean gestational age among the study group was 38.4 weeks ±2.4 (mean ± SD) which is not markedly different from the reference group’s, 38.9 weeks ±1.9 (mean ± SD). Figure 3 shows the comparison of gestational age at the time of delivery between advanced age mothers and adult mothers. Similarly, the mean birth weight of babies born from advanced age mothers was 3069.7 g ± 683.2 (mean ± SD) while babies born from adult mothers had a mean birth weight of 3160 g ± 499.1 (mean ± SD). The magnitude of adverse perinatal outcomes is shown in Table 3.

**Table 3:** magnitude of adverse perinatal outcomes of advanced and adult mothers, Ayder Comprehensive Specialized Hospital, Northern Ethiopia, 2017/18 (*n* = 752)

Perinatal outcomes		Maternal age					
		20–34 years		35+ years		Total	
		*N*	%	*N*	%	*N*	%
Perinatal Death	No	364	96.8	338	89.9	702	93.4
	Yes	12	3.2	38	10.1	50	6.6
Low Fifth Minute Apgar Score	No	354	94.4	288	76.6	643	91.1
	Yes	10	2.4	54	14.4	63	8.9
Congenital Anomalies	No	374	99.5	371	98.7	745	99.1
	Yes	2	0.5	5	1.3	7	0.9
Gestational Age	Pre-term	10	2.7	42	11.3	52	7.0
	Term	347	93.3	312	84.1	659	88.7
	Post term	15	4.0	17	4.6	32	4.3
Birth Weight	Low birth weight	20	5.4	66	17.8	86	11.6
	Normal birthweight	347	93.3	295	79.5	642	86.4
	Macrosomia	5	1.3	10	2.7	15	2.0

### Association of obstetrical outcomes with maternal age

This study encompassed 14 outcome variables. Age was significantly associated with pregnancy-induced hypertension, antepartum hemorrhage, mode of delivery, perinatal death, pre-term delivery, low birthweight, and low fifth minute Apgar score. Amniotic fluid disorders, premature rupture of membranes and post-term pregnancy were not found to have a significant association with any of the independent variables. Gestational DM, postpartum hemorrhage, macrosomia, and congenital anomalies were not analyzed using logistic regression because the estimated count of cells was less than 10.

Advanced age mothers were 4.15 times more likely to get their pregnancy complicated by pregnancy-induced hypertension than the reference group (AOR 4.15, (95% CI 2.272–7.575), *p* <  0.001). Besides APH was 2.54 times more likely to happen among the study group than the reference group (AOR 2.54 (95% CI 1.32–4.91), *P* = 0.005). Furthermore, advanced age mothers were 2.7 times more probable to undergo cesarean section than their adult counters (AOR 2.722, (95% CI 1.777–4.170), *p* < 0.001). The regression table is shown in Table 4.

**Table 4:** Bivariate and Multivariate analyses of adverse obstetrical outcomes with maternal age, Ayder Comprehensive Specialized Hospital, Northern Ethiopia, 2017/18 (*n* = 752)

Pregnancy-induced hypertension^1^					
Age group	No, n (%)	Yes, n (%)	COR (95% CI)	AOR (95% CI)	*P* value
20–34 years	355 (94.4%)	21 (5.6%)	1	1	< 0.001
≥ 35 years	310 (82.4%)	66 (17.6%)	3.599 (2.152–6.018)	4.149 (2.272–7.575) *	
Antepartum hemorrhage^1^					
	No, n (%)	Yes, n (%)	COR (95% CI)	AOR (95% CI)	*P* value
20–34 years	358 (95.2%)	18 (4.8%	1	1	0.005
≥ 35 years	318 (84.6%)	58 (15.4%)	3.628 (2.093–6.287)	2.545 (1.318–4.915) *	
Mode of Delivery^1^					
	Vaginal delivery	Cesarean delivery	COR (95% CI)	AOR (95% CI)	*P* value
20–34 years	303 (80.6%)	73 (19.4%)	1	1	< 0.001
≥ 35 years	253 (67.3%)	123 (32.7%)	2.018 (1.445–2.819)	2.722 (1.777–4.170) *	
Premature Rupture of Membrane^1^					
Age group	No, n (%)	Yes, n (%)	COR (95% CI)	AOR (95% CI)	*P* value
20–34 years	319 (84.8%)	57 (15.2%)	1	–	0.533
≥ 35 years	325 (86.4%)	51 (13.6%)	0.878 (0.584–1.321)	–	
Amniotic Fluid disorders^1^					
Age group	No, n (%)	Yes, n (%)	COR (95% CI)	AOR (95% CI)	*P* value
20–34 years	360 (95.7%)	16 (4.3%)	1	–	0.320
≥ 35 years	354 (94.1%)	22 (5.9%)	1.398 (0.722–2.707)	–	

### Association of Perinatal outcomes with maternal age

This study also found that perinatal death was associated with maternal age in which advanced age mothers were 2.54 times more likely to encounter perinatal death than adult pregnancy (AOR 2.54, 95% CI (1.141–5.635), *p* = 0.022). Pre-term delivery, low birth weight and low fifth minute Apgar score were also found to be significantly associated with maternal age. Babies born from advanced age mothers were 3.62 times more likely to be born prematurely (AOR 3.622, 95% CI (1.469–8.930), *p* = 0.005), 3.14 times more probable to be born with low birth weight (AOR 3.137, 95% CI (1.324–7.433), *p* = 0.009) and 7.51 times more likely to have low fifth minute Apgar score (AOR 7.507, 95% CI (3.134–17.98), p < 0.001) than babies born from mothers aged 20–34 years old. The regression table is shown in Table 5.

**Table 5:** Bivariate and Multivariate analyses of adverse perinatal outcomes with maternal age, Ayder Comprehensive Specialized Hospital, Northern Ethiopia, 2017/18 (*n* = 752)

Perinatal death^2^					
Age group	No, *n* (%)	Yes, *n* (%)	COR (95% CI)	AOR (95% CI)	*P* value
20–34 years	364 (96.8)	12 (3.2)	1	1	0.022
≥35 years	338 (89.9)	38 (10.1)	3.410 (1.753–6.636)	2.536 (1.141- 5.635) *	
Low fifth minute Apgar score^3≠^					
Age group	No, n (%)	Yes, n (%)	COR (95% CI)	AOR (95% CI)	*P* value
20–34 years	354 (94.4)	10 (2.4)	1	1	< 0.001
≥35 years	288 (76.6)	54 (14.4)	6.637 (3.591–15.234)	7.507 (3.134- 17.98) *	
Preterm^2≠^					
Age group	No, *n* (%)	Yes, *n* (%)	COR (95% CI)	AOR (95% CI)	*P* value
20–34 years	362 (96.3)	10 (2.7)	1	1	0.005
≥35 years	329 (87.5)	42 (11.2)	4.621 (2.282- 9.359)	3.622 (1.469- 8.930) *>	
Post term^2≠^					
Age group	No, n (%)	Yes, n (%)	COR (95% CI)	AOR (95% CI)	*P* value
20–34 years	357 (94.9)	15 (4.0)	1		0.751
≥35 years	354 (94.1)	17 (4.5)	1.143 (0.562- 2.324)	–	
Low birthweight^2≠^					
Age group	No, n (%)	Yes, n (%)	COR (95% CI)	AOR (95% CI)	*P* value
20–34 years	362 (96.3)	10 (2.7)	1	1	0.009
≥35 years	329 (87.5)	42 (11.2)	4.621 (2.282–9.359)	3.137 (1.324–7.433) *	

*n* (number), *%* (percent)

^**≠**^among 50 perinatal deaths, nine of them were intrauterine fetal death in which birth weight and weeks of gestation were not registered, four of them were early neonatal death and the rest were stillbirth. Moreover, Apgar score was not documented

* Significantly associated

^1^ The dependent variable was regressed with maternal age, residence, number of ANC visits, malpresentation, gravidity and bad obstetric history

^2^ The dependent variable was regressed with maternal age, residence, number of ANC visits, malpresentation, gravidity, bad obstetric history, PIH, APH, PROM & AF disorders

^3^ The dependent variable was regressed with maternal age, residence, number of ANC visits, malpresentation, gravidity, bad obstetric history, preterm delivery, low birth weight, PIH, APH, PROM & AF disorders

Logistic regression was not run because the estimated count of cells is less than 10 in variables such as Congenital anomalies, Post-partum hemorrhage, Macrosomia, and Gestational DM. The logistic regression tables showing the association of all independent variables with the outcome variables are attached as Additional files (Additional file 1: Table S1, Table S2, Table S3, Table S4, Table S5, Table S6, Table S7, Table S8, Table S9, Table S10).

## Discussion

This study has indicated that advanced maternal age is an important risk factor for many adverse obstetrical and perinatal outcomes. One of the main findings of this study was the association of maternal age with pregnancy-induced hypertension in which advanced age mothers were four times more likely to encounter pregnancy-induced hypertension than their adult counterparts (OR 4.15, (95% CI 2.272–7.575), *p* < 0.001). This result is congruent with the study done in Malaysia and UK with an odds ratio of 5 (AOR 5, 95% CI 1.95–12.65) and 1.5 (AOR, 1.49 (95% CI, 1.22–1.82); *P* < 0.001), respectively [10, 11]. Moreover, studies from Saudi Arabia and Turkey also stated that there was a statistically significant difference in the incidence of PIH between advanced aged and adult mothers [12, 13]. This similarity might be mainly attributed to the fact that endothelial response to vasodilators diminishes as mothers get older [14]. Nevertheless, a study conducted in India contradicts this study as it claimed that PIH has no significant association with maternal age [15]. This can be accounted for by the difference in sample size.

In addition, maternal age was found to be significantly associated with APH (OR 2.54 (95% CI 1.32–4.91), *P* = 0.005) in this study. This finding is supported by studies done in Amman Jordan [16]. This could be ascribed to increased gravidity in advanced age mothers which in turn make them at greater risk of having placenta Previa [14]. In contrast, studies from Turkey and India showed no significant association of maternal age with APH [12, 15]. This could be accounted for by differences in the setup of the study area as this study had been conducted in a tertiary hospital which mainly provides services to referral cases from the peripheral health institutions.

Another finding of this study was the association of maternal age with the mode of delivery. This study found that advanced age mothers were nearly 3 times more likely to have their babies born via cesarean section than their adult counterparts (OR 2.7, *p* < 0.001). This is in line with studies done in India (OR 2.4), Malaysia (OR 2.21) and UK (OR 1.95) [10, 11, 15]. This might be due to the fact that the proportion of malpresentation and bad obstetric history was higher in advanced aged mothers. On the other hand, mothers with bad obstetric history would rather undergo elective cesarean section because it is believed to be safer than vaginal delivery. Secondly, pregnancy complications, such as PIH and APH were commonly seen in advanced age mothers and that cesarean section could be considered for maternal reasons. Lastly, this could be due to the study area taking into consideration that this hospital is a teaching hospital for obstetrician cohorts.

In addition to these findings, this study also revealed that maternal age was not found to be a major risk factor for gestational DM, amniotic fluid disorders, PROM and PPH. However, studies from Malaysia, Saudi Arabia, the UK, and South Korea concluded that advanced maternal age was a major risk factor for gestational DM [10, 11, 13, 17]. This may be accounted for by differences in socio-economic status wherein pre-pregnancy body mass index could be affected. Paradoxically, a study in India also concluded that age was a major risk factor for PPH and amniotic fluid disorders. This could be attributed to a difference in sample size. However, this Indian study also claimed that age doesn’t have a significant association with PROM [15]. Similarly, a study in Jordan also stated that age was not found to be associated with PPH [16]. This similarity might be due to under-diagnosis of PPH.

### Perinatal outcomes

The findings of this study showed that maternal age has a significant association with preterm delivery, low birth weight, low fifth minute Apgar score, and perinatal death. In this study, advanced age mothers were almost four times more likely to have premature babies than adult mothers. This result is congruent with studies done in South Africa **(**OR = 1.37, *p* = 0.041**)**, Brazil (OR 1.66, *p* < 0.001) and South Korea (OR: 1.4 and 1.8 for 35–39 years & > 40 years, respectively, p < 0.001) [4, 17, 18]. On top of this, a multi-country assessment undertaken by WHO also is in line with this result (OR 1.2, 1.4 and 1.3 for women aged 35–39, 40–45 and ≥ 45 years old, respectively) [2]. This might be attributed to iatrogenic prematurity and to the fact that pregnancy complications are more abundant in this age group. Nevertheless, this finding is contradicted by the studies done in Malaysia and the UK which showed no association of maternal age with preterm delivery [10, 11]. This might be due to socio-economic differences.

This study also showed that maternal age was a major risk factor for low birth weight (OR 3.14, *p* = 0.009) which is supported by studies conducted in Brazil (OR 1.83 *p <* 0.001), UK (OR 1.46, *p <* 0.001) and South Africa (OR 1.67 *p* < 0.001) (20, 25, 27). Alongside this, a multi-country assessment involving 29 middle- and low-income countries also concluded that advanced maternal age was a risk factor for low birth weight (OR 1.1, 1.4 and 1.2 for women aged 35–39, 40–45 and ≥ 45 years old, respectively) [2]. This similarity could probably be due to the abundance of obstetrical complications in advanced age pregnancy and iatrogenic prematurity accompanying these adverse pregnancy complications. However, the finding of this study is inconsistent with studies done in Malaysia and Jordan which showed no significant association between maternal age and low birth weight [10, 16]. This may be accounted for by a difference in composition of the sample size. Study group and reference group in this study are proportional where as in the former studies, they were not proportionally enrolled.

Babies of advanced age mothers are almost 3 times more likely to die up to the first week of life than babies of the reference group. This study is compatible with studies done in Australia (OR 2.42, *p* < 0.001) and with the multi-country assessment held across the world (OR 1.4, 1.7 and 1.9 for women aged 35–39, 40–45 and ≥ 45 years old, respectively) [2, 19]. This might be because adverse pregnancy outcomes like PIH and APH are higher in this age group. In addition to this, it could also be due to socioeconomic characteristics of the study participants wherein prenatal visits are not attained optimally and obstetrical care services are being provided poorly. Notwithstanding this, studies from the UK and South Korea failed to support this result in which they reported there was no association of perinatal death with advanced maternal age pregnancy [11, 17]. This could primarily be due to the quality of obstetrical care services being provided.

This study also revealed that advanced age mothers are 7.5 times more probable to have their babies born with a low fifth minute Apgar score than adult mothers. This is in line with the studies done in Turkey, India, and Brazil [12, 15, 18]. Furthermore, it is also supported by multi-country assessment held in 29 countries [2]. This could be confounded by iatrogenic prematurity and its related complications. On the contrary, Jordan and Malaysian studies showed no association between low fifth minute Apgar score and advanced maternal age pregnancy [10, 16]. This could be due to a difference in sample size.

This study also elucidated that maternal age does not have a statistically significant association with macrosomia and post-term pregnancy which is compatible with study done in the UK [11]. However, a study in Brazilian stated that advanced maternal age was associated with macrosomia (OR 1.22) and post-term pregnancy (OR 1.09) [18]. This could be attributed to a difference in sample size and study design. Lastly, this study’s finding showed no association between congenital anomalies and maternal age which is contradicted by the study done in South Korea (OR 12.3) [19]. This variation could primarily be ascribed to a difference in sample size. In addition, genetic and environmental factors could also have a role in the pathogenesis of congenital anomalies.

## Conclusions

This study affirmed that advanced maternal age is significantly associated with adverse obstetrical outcomes like pregnancy-induced hypertension and antepartum hemorrhage. Cesarean delivery was also tremendously increased in those mothers. On top of this, advanced maternal age pregnancy was also found to be a major risk factor for preterm delivery, low birth-weight, low fifth minute Apgar score and perinatal death. Therefore, it is better for health care providers to counsel couples, who seek to have a child in their later ages, about the risks of advanced maternal age pregnancy. In addition, health care workers need to give emphasis on how to improve advanced age mothers’ health through the utilization of contraception in order to reduce pregnancy in this age group.

## Supplementary information

**Additional file 1: Table S1.** Logistic regression table showing association of independent variables with pregnancy induced hypertension. **Table S2.** Logistic regression table showing association of independent variables with antepartum hemorrhage. **Table S3.** Logistic regression table showing association of independent variables with cesarean delivery. **Table S4.** Logistic regression table showing association of independent variables with Preterm Delivery. **Table S5.** Logistic regression table showing association of independent variables with Low birth weight. **Table S6.** Logistic regression table showing association of independent variables with perinatal death. **Table S7.** Logistic regression table showing association of independent variables with low fifth minute Apgar score. **Table S8.** Logistic regression table showing association of independent variables with amniotic fluid disturbances, **Table S9.** Logistic regression table showing association of independent variables with premature rupture of membranes. **Table S10.** Logistic regression table showing association of independent variables with post-term pregnancy

## Data Availability

The datasets generated and/or analyzed during the current study are available from the corresponding author upon reasonable request. All necessary materials are included in the manuscript as separate files.

## Abbreviations

AF: Amniotic Fluid
AMA: Advanced Maternal Age
AOR: Adjusted Odds Ratio
APH: Antepartum Hemorrhage
DM: Diabetes Mellitus
GDM: Gestational Diabetes Mellitus
GTDs: Gestational Trophoblastic Diseases
HMIS: Health Management Information System
HTN: Hypertension
IUFD: Intrauterine Fetal Death
LBW: Low Birth Weight
MD: Maternal Death
MNM: Maternal Near Miss
NICU: Neonatal Intensive Care Unit
PI: Principal Investigator
PIH: Pregnancy Induced Hypertension
PPH: Postpartum Hemorrhage
PROM: Premature Rupture of Membrane
SMO: Severe Maternal Outcome
SOGC: Society of Obstetrics and Gynecology Of Canada
UK: United Kingdom
Vs: Versus
