# Supplementary material for: Advanced maternal age pregnancy and its adverse obstetrical and perinatal outcomes in Ayder comprehensive specialized hospital, Northern Ethiopia, 2017: a comparative cross-sectional study
Source: BMC Pregnancy Childbirth. 2020 Jan 30;20:60. doi: 10.1186/s12884-020-2740-6 (PMC6993443; doi:10.1186/s12884-020-2740-6)
Supplement: Supplementary file 1 — Additional file 1: Table S1. Logistic regression table showing association of independent variables with pregnancy induced hypertension. Table S2. Logistic regression table showing association of independent variables with antepartum hemorrhage. Table S3. Logistic regression table showing association of independent variables with cesarean delivery. Table S4. Logistic regression table showing association of independent variables with Preterm Delivery. Table S5. Logistic regression table showing association of independent variables with Low birth weight. Table S6. Logistic regression table showing association of independent variables with perinatal death. Table S7. Logistic regression table showing association of independent variables with low fifth minute Apgar score. Table S8. Logistic regression table showing association of independent variables with amniotic fluid disturbances, Table S9. Logistic regression table showing association of independent variables with premature rupture of membranes. Table S10. Logistic regression table showing association of independent variables with post-term pregnancy [file 12884_2020_2740_MOESM1_ESM.docx]

**Table S1: Logistic regression table showing association of independent variables with pregnancy induced hypertension**

| Independent variables | Category | Crude OR | Adjusted OR | P value |
| --- | --- | --- | --- | --- |
| Age | 20 – 34 years | 1 |  | <0.001 |
|  | ≥35 years | 3.599 (2.152 - 6.018) | **4.149 (2.272 - 7.575)^*^** |  |
| Residence | Urban | 1 |  | 0.26 |
|  | Rural | 1.325 (0.838 - 2.094) | 1.340 (0.800 - 2.243) |  |
| Bad obstetric history | No | 1 | - | 0.33 |
|  | Yes | 0.674 (0.319 - 1.427) |  |  |
| Number of ANC visits | Four and above | 1 |  | 0.084 |
|  | Less than four | 0.670 (.336 - 1.336) | 0.518 (0.246 - 1.093) |  |
| Gravidity | Primigravida | 1 | 1 |  |
|  | Multi gravida | 0.338 (0.119 - 0.959) | 0.810 (0.408 - 1.605) | .545 |
|  | Grand multi gravida | 0.513 (0.199 - 1.322) | 0.614 (0.253 - 1.489) | .281 |
|  | Great grand multi gravida | 0.611 (0.219 - 1.705 | 1.055 (0.311 - 3.574) | .932 |
| Malpresentation | No | 1 | - | 0.65 |
|  | Yes | 1.247 (.482 - 3.226) |  |  |

**Table S2: Logistic regression table showing association of independent variables with antepartum hemorrhage**

| Independent variables | Category | Crude OR | Adjusted OR | P value |
| --- | --- | --- | --- | --- |
| Age | 20 – 34 years | 1 | 1 | 0.005 |
|  | ≥35 years | 3.628 (2.093 - 6.287) | **2.545 (1.318 - 4.915)^*^** |  |
| Residence | Urban | 1 | 1 | 0.004 |
|  | Rural | 3.547 (2.172 - 5.793) | **2.281 (1.299 - 4.004)^*^** |  |
| Bad obstetric history | No | 1 | 1 | 0.212 |
|  | Yes | 2.629 (1.323 - 5.225) | 1.652 (0.751 - 3.631) |  |
| Number of ANC visits | Four and above | 1 | 1 | <0.001 |
|  | Less than four | 4.664 (2.801 - 7.766) | **3.227 (1.834 - 5.679)^*^** |  |
| Gravidity | Primigravida | 1 | 1 |  |
|  | Multi gravida | 3.815 (1.479 - 9.845) | 2.245 (0.821 - 6.142) | .115 |
|  | Grand multi gravida | 7.266 (2.674 - 19.746) | 1.767 (0.538 - 5.802) | .348 |
|  | Great grand multi gravida | 18.947 (5.863 - 61.233) | 2.553 (0.607 - 10.743) | .201 |
| Malpresentation | No | 1 | - | 0.14 |
|  | Yes | 0.338 (0.081 - 1.419) |  |  |

**Table S3: Logistic regression table showing association of independent variables with cesarean delivery**

| Independent Variables | Category | Crude OR | Adjusted OR | P value |
| --- | --- | --- | --- | --- |
| Age | 20 – 34 years | 1 | 1 | <0.001 |
|  | ≥35 years | 2.018 (1.445 - 2.819) | **2.722 (1.777 - 4.170)^*^** |  |
| Residence | Urban | 1 | - | 0.257 |
|  | Rural | 1.216 (0.867 - 1.706) |  |  |
| Bad obstetric history | No | 1 |  | <0.001 |
|  | Yes | 3.275 (1.894 - 5.662) | **3.303 (1.763 - 6.191)^*^** |  |
| Number of ANC visits | Four and above | 1 | - | 0.529 |
|  | Less than four | 0.863 (0.545 - 1.366) |  |  |
| Gravidity | Primigravida | 1 |  |  |
|  | Multi gravida | 0.336 (0.151 - 0.747) | 1.657 (0.611 - 4.496) | 0.321 |
|  | Grand multi gravida | 0.296 (0.138 - 0.635) | 0.845 (0.344 - 2.078) | 0.714 |
|  | Great grand multi gravida | 0.327 (0.143 - 0.748) | 0.458 (0.180 - 1.166) | 0.101 |
| Malpresentation | No | 1 | 1 | <0.001 |
|  | Yes | 6.893 (3.762 - 12.629) | **6.696 (3.531 - 12.701)^*^** |  |
| Post term | No | 1 | - | 0.812 |
|  | Yes | 0.912 (0.415 - 2.006) |  |  |

**Table S4: Logistic regression table showing association of independent variables with Preterm Delivery**

| Independent Variables | Category | Crude OR | Adjusted OR | P value |
| --- | --- | --- | --- | --- |
| Age | 20 – 34 years | 1 | 1 | 0.005 |
|  | ≥35 years | 4.621 (2.282 - 9.359) | **3.622 (1.469 - 8.930)^*^** |  |
| Residence | Urban | 1 | 1 | <0.001 |
|  | Rural | 1.216 (0.867 - 1.706) | **4.390 (2.131 - 9.045)^*^** |  |
| Bad obstetric history | No | 1 |  |  |
|  | Yes | 1.364 (0.519 - 3.583) | - |  |
| Number of ANC visits | Four and above | 1 | 1 | 0.592 |
|  | Less than four | 2.368 (1.254 - 4.473) | 1.260 (0.541 - 2.933) |  |
| Gravidity | Primigravida | 1 |  |  |
|  | Multi gravida | 1.936 (0.780 - 4.803) | .681 (0.235 - 1.978) | .481 |
|  | Grand multi gravida | 4.330 (1.645 - 11.402) | 0.653 (0.180 - 2.365) | .516 |
|  | Great grand multi gravida | 6.146 (1.741 - 21.690) | 0.435 (0.090 - 2.114) | .302 |
| Pregnancy induced hypertension | No | 1 | 1 | <0.001 |
|  | Yes | 3.485 (1.824 - 6.661) | **4.741 (2.148 - 10.463)^*^** |  |
| Antepartum hemorrhage | No | 1 | 1 | <0.001 |
|  | Yes | 5.508 (2.902 - 10.451) | **7.112 (3.078 16.435)^*^** |  |
| Premature rupture of membrane | No | 1 | 1 | <0.001 |
|  | Yes | 3.581 (1.940 - 6.611) | **8.547 (3.917 - 18.653)^*^** |  |
| AF disorders | No | 1 | 1 | 0.072 |
|  | Yes | 2.686 (1.069 - 6.752) | - 1. .909 - 7.732) |  |

**Table S5: Logistic regression table showing association of independent variables with Low birth weight**

| Independent Variables | Category | Crude OR | Adjusted OR | P value |
| --- | --- | --- | --- | --- |
| Age | 20 – 34 years | 1 | 1 | 0.009 |
|  | ≥35 years | 4.621 (2.282 - 9.359) | **3.137 (1.324 - 7.433)^*^** |  |
| Residence | Urban | 1 | 1 | <0.001 |
|  | Rural | 4.915 (2.669 - 9.049) | **3.916 (1.956 - 7.841)^*^** |  |
| Bad obstetric history | No | 1 | - | 0.441 |
|  | Yes | 1.364 (0.519 - 3.583) |  |  |
| Number of ANC visits | Four and above | 1 | 1 | 0.728 |
|  | Less than four | 2.368 (1.254 - 4.473) | 1.149 (0.525 - 2.513) |  |
| Gravidity | Primigravida | 1 | 1 | 0.569 |
|  | Multi gravida | 1.936 (0.780 - 4.803) | 0.741 (0.264 - 2.077) |  |
|  | Grand multi gravida | 4.330 (1.645 - 11.402) | 0.721 (0.211 - 2.464) | 0.602 |
|  | Great grand multi gravida | 6.146 (1.741 - 21.690) | 0.570 (0.120 - 2.719) | 0.481 |
| Pregnancy induced hypertension | No | 1 | 1 | 0.002 |
|  | Yes | 3.485 (1.824 - 6.661) | **3.321 (1.582 - 6.973)^*^** |  |
| Antepartum hemorrhage | No | 1 | 1 | <0.001 |
|  | Yes | 5.508 (2.902 - 10.451) | **4.454 (2.106 - 9.419)^*^** |  |
| Premature rupture of membrane | No | 1 | 1 | 0.112 |
|  | Yes | 3.581 (1.940 - 6.611) | 2.731 (0.931 – 4.179) |  |
| AF disorders | No | 1 | 1 | 0.177 |
|  | Yes | 2.686 (1.069 - 6.752) | 2.048 (0.723 - 5.803) |  |

**Table S6: Logistic regression table showing association of independent variables with perinatal death**

| Independent Variables | Category | Crude OR | Adjusted OR | P value |
| --- | --- | --- | --- | --- |
| Age | 20 – 34 years | 1 | 1 | 0.022 |
|  | ≥35 years | 2.011 (1.060 - 3.816) | **2.536 (1.141 - 5.635)^*^** |  |
| Residence | Urban | 1 | 1 |  |
|  | Rural | 2.190 (1.188 - 4.039) |  |  |
| Bad obstetric history | No | 1 |  |  |
|  | Yes | 2.989 (1.317 - 6.780) | - |  |
| Number of ANC visits | Four and above | 1 | 1 | 0.240 |
|  | Less than four | 3.038 (1.574 - 5.863) | 1.561 (0.742 - 3.283) |  |
| Gravidity | Primigravida | 1 | 1 | 0.671 |
|  | Multi gravida | 1.623 (0.644 - 4.091) | .812 (0.312 - 2.117) |  |
|  | Grand multi gravida | 4.151 (1.578 - 10.917) | 1.319 (0.411 - 4.236) | 0.642 |
|  | Great grand multi gravida | 1.065 (0.124 - 9.185) | 0.547 (0.098 - 3.070) | 0.493 |
| Malpresentation | No | 1 |  | 0.527 |
|  | Yes | 0.627 (0.147 - 2.665) |  |  |
| Pregnancy induced hypertension | No | 1 |  | 0.579 |
|  | Yes | 1.266 (0.551 - 2.909) |  |  |
| Antepartum hemorrhage | No | 1 | 1 | <0.001 |
|  | Yes | 5.035 (2.627 - 9.650) | **3.444 (1.681 - 7.056)^*^** |  |
| Premature rupture of membrane | No | 1 |  | 0.940 |
|  | Yes | 0.969 (0.424 - 2.213) |  |  |
| AF disorders | No | 1 |  | 0.726 |
|  | Yes | 0.771 (0.180 - 3.298) |  |  |
| Mode of delivery | Vaginal | 1 |  | 0.499 |
|  | Cesarean delivery | 1.269 (0.636 - 2.529) |  |  |

**Table S7: Logistic regression table showing association of independent variables with low fifth minute Apgar score**

| Independent Variables | Category | Crude OR | Adjusted OR | P value |
| --- | --- | --- | --- | --- |
| Age | 20 – 34 years | 1 | 1 | <0.001 |
|  | ≥35 years | 6.637 (3.591 -15.234) | **7.50 (3.134 - 17.98)^*^** |  |
| Residence | Urban | 1 | 1 | 0.508 |
|  | Rural | 2.236 (1.328 - 3.764) | 1.228 (0.668 - 2.256) |  |
| Bad obstetric history | No | 1 | - | 0.489 |
|  | Yes | 1.526 (0.461 - 5.054) |  |  |
| Number of ANC visits | Four and above | 1 | 1 | 0.241 |
|  | Less than four | 1.966 (1.056 - 3.660) | 1.553 (0.744 - 3.240) |  |
| Gravidity | Primigravida | 1 | 1 | 0.342 |
|  | Multi gravida | 0.391 (0.115 - 1.325) | - |  |
|  | Grand multi gravida | 0.521 (0.170 - 1.597) |  | 0.641 |
|  | Great grand multi gravida | 1.030 (0.318 - 3.343) |  | 0.960 |
| Malpresentation | No | 0.488 (0.218 - 1.090) | 0.687 (0.275 - 1.715) | 0.421 |
|  | Yes | 1 | 1 |  |
| Pregnancy induced hypertension | No | 1 | 1 | 0.249 |
|  | Yes | 3.080 (1.651 - 5.744) | 1.532 (0.742 - 3.163) |  |
| Antepartum hemorrhage | No | 1 | 1 | 0.450 |
|  | Yes | 1.855 (0.868 - 3.966) | 0.699 (0.277 - 1.768) |  |
| Premature rupture of membrane | No | 1 | - | 0.634 |
|  | Yes | .829 (0.383 - 1.795) |  |  |
| AF disorders | No | 1 | 1 | 0.639 |
|  | Yes | 2.151 (0.859 - 5.384) | 1.285 (0.451 - 3.665) |  |
| Mode of delivery | Vaginal | 1 | 1 | 0.037 |
|  | Cesarean delivery | 2.791 (1.649 - 4.722) | **1.912 (1.039 - 3.518)*** |  |
| Preterm delivery | No | 1 | 1 | <0.001 |
|  | Yes | 7.825 (4.006 - 15.283) | **4.962 (2.294 - 10.733)^*^** |  |

**Table S8: Logistic regression table showing association of independent variables with amniotic fluid disturbances**

| Independent variables | Category | Crude OR | Adjusted OR | P value |
| --- | --- | --- | --- | --- |
| Age | 20 – 34 years | 1 |  | 0.320 |
|  | ≥35 years | (0.722 – 2.707) |  |  |
| Residence | Urban | 1 |  | 0.310 |
|  | Rural | 1.410 (0.727 - 2.734) |  |  |
| Bad obstetric history | No | 1 | - | 0.582 |
|  | Yes | 0.666 (0.156 – 2.838) |  |  |
| Number of ANC visits | Four and above | 1 |  | 0.660 |
|  | Less than four | 0.806 (.308 – 2.108) |  |  |
| Gravidity | Primigravida | 1 |  |  |
|  | Multi gravida | 0.732 (0.339 - 1.583) |  | .428 |
|  | Grand multi gravida | 1.167(0.469 - 2.901) |  | .740 |
| Malpresentation | No | 1 | - | 0.682 |
|  | Yes | .738- (0.173 - 3.153) |  |  |
| Post term | No | 1 |  | 0.323 |
|  | Yes | 1.998 (0.580 - 6.879) | 1.876 (0.539 - 6.538) |  |

**Table S9: Logistic regression table showing association of independent variables with premature rupture of membranes**

| Independent variables | Category | Crude OR | Adjusted OR | P value |
| --- | --- | --- | --- | --- |
| Age | 20 – 34 years | 1 |  | .533 |
|  | ≥35 years | .878 (.584 - 1.321) |  |  |
| Residence | Urban | 1 |  | .631 |
|  | Rural | 1.112 (.720 - 1.717) |  |  |
| Bad obstetric history | No | 1 | - | .477 |
|  | Yes | 1.296 (.634 - 2.650) |  |  |
| Number of ANC visits | Four and above | 1 |  | .073 |
|  | Less than four | .569 (.295 – 1.098) | 1.889 (.941 - 3.790) |  |
| Gravidity | Primigravida | 1 |  |  |
|  | Multi gravida | .876 (.539 - 1.423) |  | .593 |
|  | Grand multi gravida | .748 (.388 - 1.443) |  | .387 |
|  | Great grand multi gravida | 1.403 (.526 - 3.747) |  | .499 |
| Malpresentation | No | 1 | - | .548 |
|  | Yes | .765 (.318 - 1.837) |  |  |

**Table S10: Logistic regression table showing association of independent variables with post-term pregnancy**

| Independent variables | Category | Crude OR | Adjusted OR | P value |
| --- | --- | --- | --- | --- |
| Age | 20 – 34 years | 1 |  | .712 |
|  | ≥35 years | 1.143 (.562 - 2.324) |  |  |
| Residence | Urban | 1 |  | .674 |
|  | Rural | .855 (.411 - 1.778) |  |  |
| Number of ANC visits | Four and above | 1 |  | .621 |
|  | Less than four | 1.309 (.450 - 3.804) |  |  |
| Gravidity | Primigravida | 1 |  |  |
|  | Multi gravida | .698 (.143 - 3.407) |  | .657 |
|  | Grand multi gravida | .482 (.104 - 2.231) |  | .351 |
|  | Great grand multi gravida | .801 (.158 - 4.072) |  | .789 |
| Malpresentation | No | 1 |  | .395 |
|  | Yes | .417 (.056 - 3.121) |  |  |
